# Change in the Lipid Transport Capacity of the Liver and Blood during Reproduction in Rats

**DOI:** 10.3389/fphys.2017.00517

**Published:** 2017-07-26

**Authors:** Yufeng Zhang, Christine Kallenberg, Hayden W. Hyatt, Andreas N. Kavazis, Wendy R. Hood

**Affiliations:** ^1^Department of Biological Science, Auburn University Auburn, AL, United States; ^2^School of Kinesiology, Auburn University Auburn, AL, United States

**Keywords:** phenotypic plasticity, post-lactation, FAT/CD36, FABPpm, FABP_c_, albumin, apolipoprotein B

## Abstract

To support the high energetic demands of reproduction, female mammals display plasticity in many physiological processes, such as the lipid transport system. Lipids support the energy demands of females during reproduction, and energy and structural demands of the developing offspring via the placenta *in utero* or milk during the suckling period. We hypothesized that key proteins supporting lipid transport in reproductive females will increase during pregnancy and lactation, but drop to non-reproductive levels shortly after reproduction has ended. We compared the relative protein levels of liver-type cytosolic fatty acid transporter (L-FABP_*c*_), plasma membrane fatty acid transporter (FABPpm), fatty acid translocase (FAT/CD36) in the liver, a key site of lipid storage and synthesis, and free fatty acid transporter albumin and triglyceride transporter [represented by apolipoprotein B (apoB)] levels in serum in reproductive Sprague-Dawley rats during late pregnancy, peak-lactation, and 1-week post-lactation as well as in non-reproductive rats. We found that all lipid transporter levels were greater in pregnant rats compared to non-reproductive rats. Lactating rats also showed higher levels of FAT/CD36 and FABPpm than non-reproductive rats. Moreover, all fat transporters also dropped back to non-reproductive levels during post-lactation except for FAT/CD36. These results indicate that fat uptake and transport capacities in liver cells are elevated during late gestation and lactation. Liver lipid secretion is up-regulated during gestation but not during lactation. These data supported the plasticity of lipid transport capacities in liver and blood during reproductive stages.

## Introduction

To meet the energetic and resource demands of reproduction, female mammals display considerable morphological and physiological flexibility. Morphological changes include an increase in the size of the uterus, development of the placenta, growth of mammary tissue, and increased capacity of the gastrointestinal tract (Speakman, 2008). Physiological changes include increased blood volume and circulating hemoglobin, up-regulation of antibody production, and change in pattern of nutrient handling and synthesis (Knobil and Neill, 2006; Zhang and Hood, 2016). These changes are typically short lived and regress shortly after gestation and/or lactation has ended.

For all mammalian species, successful reproduction requires that a mother's pattern of nutrient delivery match her offspring's requirement for growth and development. In general, the demands of early tissue accretion and development *in utero* are met with glucose and amino acids (Battaglia and Meschia, 1978; Brett et al., 2014). The amount of lipid that is transferred across the placenta is typically low relative to the other macronutrients (Girard et al., 1992). Nevertheless, lipids play a critical role in nervous system development (Girard et al., 1992; Brett et al., 2014). During lactation, several mammalian taxa display a short spike in fat transfer via colostrum followed by an immediate drop in the fat content of mature milk (Oftedal, 1984; Nicholas and Hartmann, 1991; Skibiel and Hood, 2013). The fat content of mature milk then remains stable or increases until the young starts consuming solid food. At the point just before pups begin consuming solid food, the percent fat in milk exceeds that of carbohydrate and protein in nearly all rodents studied to date (Oftedal, 1984; Oftedal and Iverson, 1995; Kunz and Hood, 2000; Skibiel and Hood, 2013; but see Hood et al., 2014). Fat is energy dense and thus, high fat milk supports rapid growth.

To meet an offspring's demand for lipid, females may alter their diet to include more lipid or more glucose to fuel lipogenesis (van Knegsel et al., 2007), store lipid as adipose for later mobilization (Havel, 1987), and alter their patterns of lipid use and handling (Kiens, 2006) to ensure that sufficient lipids are available for transfer to the offspring via the placenta or in milk. The liver plays a vital role in lipid metabolism because it is responsible for lipid degradation, modification, and lipid synthesis (Atshaves et al., 2010; Price, 2016). Despite its central role in supporting the demands of reproduction, our knowledge of how lipid handling by the liver changes in response to reproduction is limited.

While several studies have investigated lipid transport in placenta and mammary glands during pregnancy (Cunningham and McDermott, 2009; Brett et al., 2014) and lactation (Spitsberg et al., 1995; Gutgesell et al., 2009; McManaman, 2014), there is little data on lipid mobilization and transport in the liver during reproduction (Besnard et al., 1995; Schlegel et al., 2012; Akbar et al., 2013). The liver produces new and modifies existing triacylglycerols (TG) and non-esterified fatty acids (NEFA; Janero et al., 1984). NEFA must be transported within the cells of the liver by L-FABP_c_ (Meunier-Durmort et al., 1996) and cross liver cell membranes via a fatty acid binding protein (FABPpm) and fatty acid translocase (FAT/CD36) before they are picked up by carrier proteins (Luiken et al., 2002; Glatz et al., 2010; Mashek, 2013). Albumin transports NEFA from adipose to the liver, while lipoproteins are responsible for secretion of TG from liver (Saifer and Goldman, 1961; Smith et al., 1978; Frayn et al., 2006). Several forms of lipoproteins exist. Very low-density lipoproteins (VLDL) are exclusively synthesized and produced in the liver. Some VLDL will convert to low-density lipoproteins (LDL) and intermediate-density lipoproteins (IDL) in the blood. All these lipoproteins carry apolipoprotein B-100 (apoB-100) as a core functional unit (Mahley et al., 1984). Because of their vital roles in lipid transport, an up- or down-regulation of L-FABP_c_, FABPpm, FAT/CD36, liver-derived lipoproteins, and/or albumin can alter the availability of fatty acids to maternal tissues and to the developing young.

In this study, we investigate processes that support lipid transport to and from and within liver during reproduction in the laboratory rat. We measured protein levels of FAT/CD36, FABPpm, and L-FABP_c_ to examine effects of reproduction on intracellular and across-membrane lipid transport in the liver. We also measured albumin and apoB-100 in blood to examine the lipid transport capacity of rats during gestation and lactation. We predicted that lipid transport capacity would be elevated during gestation and lactation, facilitating adipose deposition in gestating females and ensuring that lipids needed for fetal development and post-natal growth are transported to the placenta and mammary glands, respectively. To our knowledge, this is the first study to concurrently measure multiple key regulatory steps of fatty acid transport as a function of gestation and lactation.

## Materials and methods

All procedures in this study were approved by the Auburn University Institutional Animal Care and Use Committee (Protocol 2014-2591).

### Animal care, experimental design, and biological sample collection

Outbred Sprague-Dawley rats (Envigo, Inc.) were used in this experiment. Rats were housed in Auburn University's Biological Research Facility in 46 × 25 × 20 cm rat cages with a standard wire hopper, and water bottle. Food (2018 Teklad Global 18% Protein Rodent Diet; Envigo, Inc.) and water were provided to all rats *ad libitum*. Rats were kept on a 14:10 h light:dark cycle to provide the appropriate light stimulus for mating. After 3 days of acclimation period, rats were randomly assigned into four groups (*n* = 8/group): non-reproductive, pregnant, lactating, and post-lactation. Reproductively mature females (~12 weeks) were housed in pairs with non-reproductive females housed with other females and other three groups (pregnant, lactation, and post-lactation groups) were paired with males for mating. Females in the pregnant group were euthanized at ~3 days before parturition (i.e., day 18 of pregnancy; estimated by relative girth). Females in the lactating and post-lactation groups had their litter size adjusted to 8 pups the first day after parturition. Animals in the lactating group were allowed to nurse their pups and were euthanized at day 15 of lactation. Animals in the post-lactation group were allowed to nurse their pups for 21 days, before the pups were removed (weaning) and the animals were euthanized 7 days later.

Rats were anesthetized with an overdose of isoflurane vapors. After the respiration of the rats slowed and were no longer responsive to a foot pinch, the animals were decapitated with a rodent guillotine. Blood was collected from the rats after decapitation in 10 ml Serum Plus Blood Collection Tubes (BD Vacutainer, Franklin Lakes, NJ, USA). Then, serum was collected by centrifuging whole blood at 1,500 × g for 10 min using IEC Centra CL2 centrifuge (Thermofisher Scientific, Waltham, MA, USA). The liver of each rat was removed and flash frozen in liquid nitrogen. Liver and serum samples were stored at −80°C for subsequent analyses.

### Western blot for liver protein expression

Approximately 100 mg of liver was homogenized on ice with a glass-glass homogenizer (Caframo; Wiarton, ON, Canada) for 10-s in 1 mL of homogenizing buffer (5 mM Tris-HCL, pH 7.4; 5 mM EDTA; Protease Inhibitor Cocktail) and centrifuged at 1,500 × g for 10 min at 4°C. The resulting supernatant (cytosolic) fraction was collected and protein content was assessed by the method of Bradford (Sigma). Equal amounts of protein were separated by using 4–12% SDS-PAGE Protein Gels. Randomly chosen sample was included on all gels to serve as a standard for detecting gel-to-gel variation. After electrophoresis, the proteins were transferred to polyvinylidene difluoride (PVDF) membranes (Amresco). Membranes were blocked for 1 h at room temperature in PBS solution containing 0.05% Tween and 5% non-fat milk. Membranes were then incubated overnight at 4°C with primary antibodies directed against the proteins of interest. Specifically, the following primary antibodies were used: FAT/CD36 (rabbit polyclonal; Thermo Fisher, Rockford, IL; 1:1,000 dilution), FABPpm (rabbit polyclonal, Thermo Fisher, Rockford, IL; 1:1,000 dilution), L-FABP (rabbit polyclonal, Thermo Fisher, Rockford, IL; 1:1,000 dilution), and α-tubulin (mouse polyclonal; GeneTex, Irvine, CA; 1: 2,000 dilution) was used as the normalizing control. Following incubation with primary antibodies, membranes were washed extensively with PBS-Tween and then incubated with secondary antibodies [anti-rabbit (1:2,000; GeneTex, Irvine, CA)]. Membranes were then developed using an enhanced chemiluminescent reagent (Amersham, Pittsburgh, PA), and band densitometry was performed through the use of a UVP Imager and associated densitometry software (UVP, LLC, Upland, CA).

### ELISA for serum protein expression

Commercially available ELISA kits were used to quantify serum albumin (Abcam; Cambridge, MA, USA) and apolipoprotein B (Cloud-Clone Corp; Houston, TX, USA). Briefly, all reagents, samples and standards were prepared according to the manufacturers' instructions and added to each well in 96-well plates provided with the kit. Each well was washed and streptavidin-HRP conjugate was added and incubated. Then, chromogen substrate was added to each well before the stop solution. The contents of the plates were then measured at a wavelength of 450 nm using the Synergy H1 Hybrid plate reader (BioTek; Winooski, VT, USA). Data from each plate were calculated using standard curve run in each plate.

### Statistics

Data are presented as means ± standard error (SE). Data for each measurement were tested using the Grubb's outlier test (Grubbs, 1969), and then analyzed using one-way ANOVA followed by Tukey *post-hoc* test if appropriate. All statistical analyses were performed with SigmaStat 3.5 (Systat Software, Inc., Point Richmond, CA, USA). We accepted statistical significance for all tests at *P* < 0.05.

## Results

### Across-membrane and intracellular lipid transport

**Figure 1:**
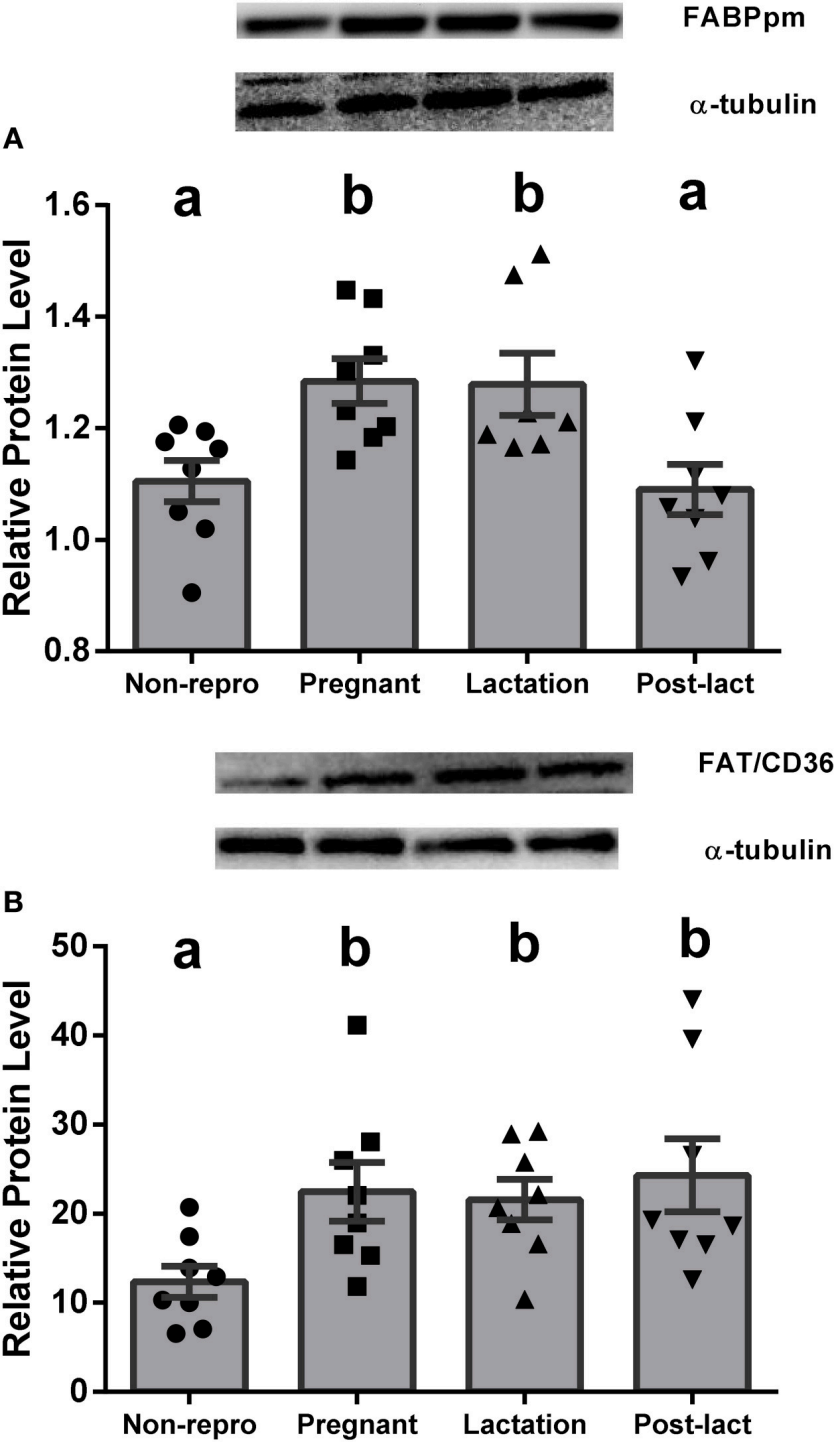
**(A)** Relative protein levels from western blot assays for plasma membrane-bound fatty acid binding protein (FABPpm) in liver of non-reproductive (non-repro, *n* = 8), pregnant (*n* = 8), lactation (*n* = 7), and post-lactation (post-lact, *n* = 8) rats. **(B)** Relative protein levels from western blot assays for fatty acyl translocase (FAT/CD36) in liver of non-reproductive control (*n* = 8), pregnant (*n* = 8), lactation (*n* = 8), and post-lactation (post-lact, *n* = 8) rats. Error bars represent SE. Bars with different letters above them are significantly different.

**Figure 2:**
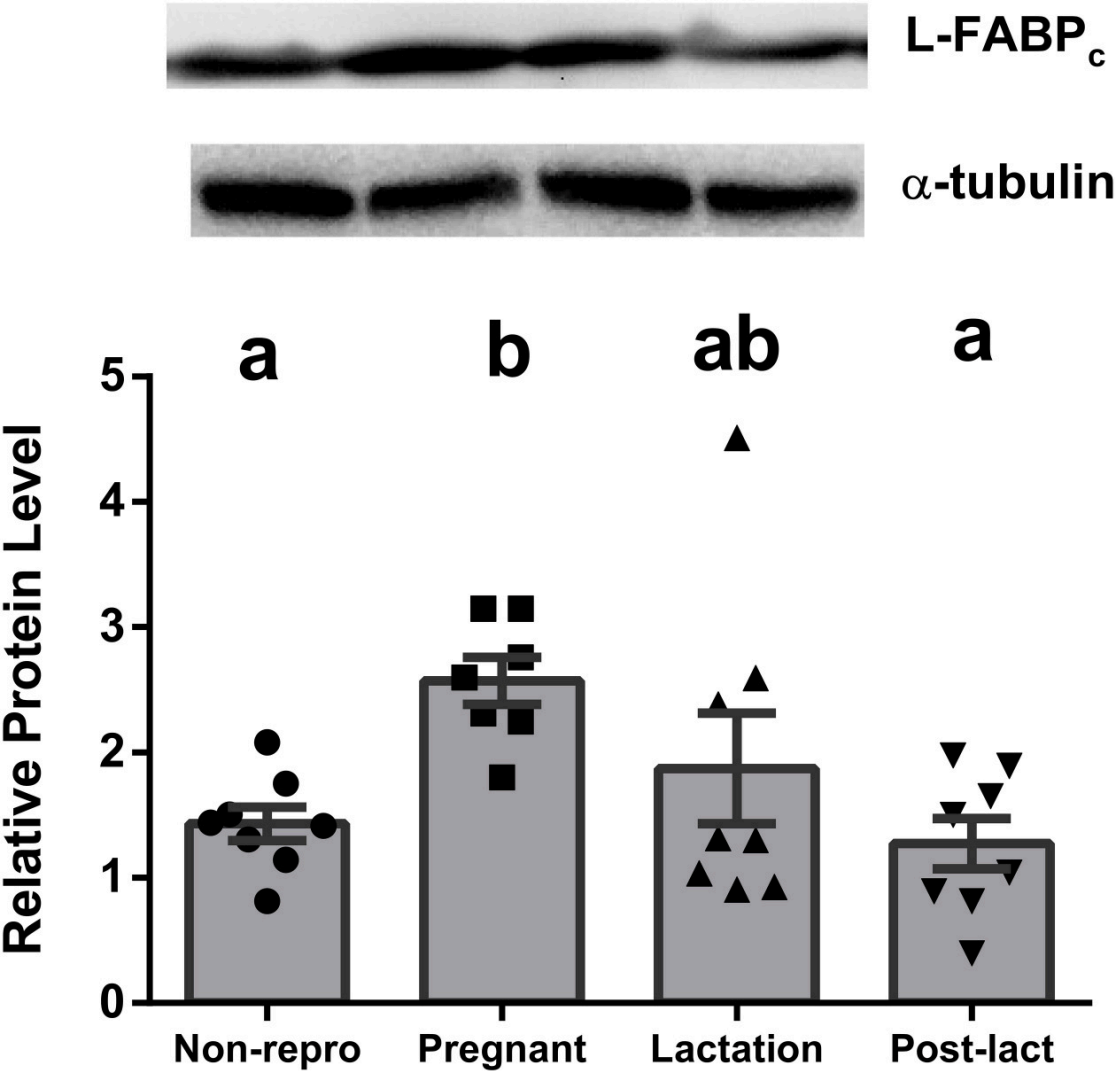
Relative protein levels from western blot assays for liver type cytosolic fatty acid binding protein (L-FABP_c_) in liver of non-reproductive (non-repro, *n* = 8), pregnant (*n* = 7), lactation (*n* = 8), and post-lactation (post-lact, *n* = 8) rats. Error bars represent SE. Bars with different letters above them are significantly different.

Liver NEFA uptake capacities were measured by protein levels of FABPpm and FAT/CD36. FABPpm protein levels in liver were significantly different between groups [*F*_(3, 27)_ = 6.40, *P* < 0.01, Figure 1A], with both pregnant and lactating rats having higher FABPpm relative to non-reproductive and post-lactation rats. FAT/CD36 protein levels were significantly higher in pregnant, lactating, and post-lactation animals compared to non-reproductive animals [*F*_(3, 28)_ = 4.815, *P* < 0.01, Figure 1B]. Liver intracellular NEFA transport capacities were represented by L-FABP_c_. L-FABP_c_ protein levels in liver were also highest during pregnancy [*F*_(3, 27)_ = 5.77, *P* < 0.01, Figure 2] and lowest in non-reproductive and post-lactating rats. Lactating rats were not significantly different from any other group.

### Lipid transport in blood

**Figure 3:**
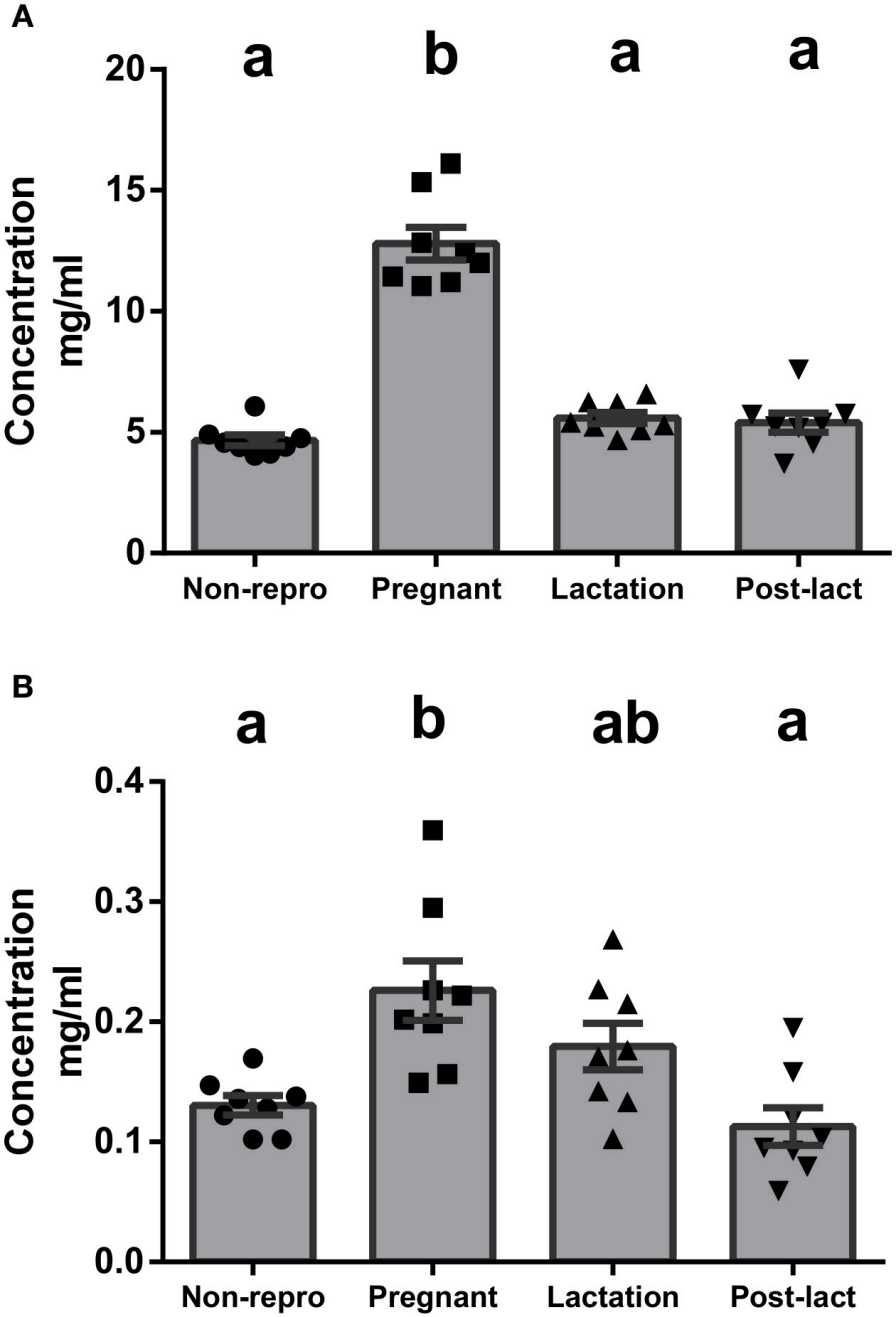
**(A)** Protein concentration from ELISA for albumin in serum of non-reproductive (non-repro, *n* = 8), pregnant (*n* = 8), lactation (*n* = 8), and post-lactation (post-lact, *n* = 8) rats. **(B)** Protein concentration from ELISA for apolipoprotein B in serum of non-reproductive control (*n* = 8), pregnant (*n* = 8), lactation (*n* = 8), and post-lactation (*n* = 8) rats. Error bars represent SE. Bars with different letters above them are significantly different.

We measured albumin to represent NEFA transport levels in blood. Albumin protein concentrations in serum were significantly different between groups [*F*_(3, 28)_ = 72.3, *P* < 0.01, Figure 3A]. Specifically, pregnant rats had significantly higher levels of circulating albumin than non-reproductive, lactating, or post-lactating rats. To measure liver lipid secretion and TG transport capacities, we measured apoB-100 protein concentration in serum. The protein levels of apoB were significantly different between groups [*F*_(3, 28)_ = 7.10, *P* < 0.01, Figure 3B]. Circulating apoB was highest during pregnancy, intermediate during lactation, and lowest in non-reproductive and post-lactating rats.

## Discussion

This study demonstrates changes that occur in the lipid uptake and transport capacities of the liver cell and TG secretion levels from liver during reproduction. Liver-derived lipoproteins (as indicated by apoB-100) that transport TGs were also high at both of these time points. In contrast, albumin, which is responsible for NEFA transport, was only high during pregnancy. Proteins that facilitate NEFA transport within liver cells (L-FAPB_c_) and across liver cell membranes (FABPpm, FAT/CD36) were up-regulated during late pregnancy and peak-lactation. These data indicate an important role for lipid uptake, transport and secretion by the liver during different stages of reproduction.

The relative concentrations of serum lipid transport proteins described herein highlight differences in the demand for lipid during pregnancy and lactation in rats, with both albumin and apoB-100 peaking during pregnancy, and albumin dropping to non-reproductive levels before peak lactation. ApoB-100 levels remain elevated during lactation, but to a lesser degree, and reach non-reproductive levels post-lactation. Elevated apoB-100 during pregnancy and lactation is consistent with studies showing increasing levels of circulating TG, total cholesterol, and total lipoproteins throughout pregnancy (Smith and Welch, 1976; Knopp et al., 1986; Parchwani and Patel, 2011; Emet et al., 2013; Pusukuru et al., 2016) and elevated apoB-100, TG, and total lipoprotein during lactation (Smith et al., 1998). Thus, the secretion of TG and its transport via lipoproteins appears to serve as a common regulatory point during pregnancy and lactation.

A number of studies have documented that peripheral tissues that normally display high rates of fatty acid utilization, such as skeletal muscle and the heart, reduce use of fatty acids during reproduction by down-regulation of fatty acid uptake and oxidation (Pedraza et al., 2001; Xiao et al., 2004; Gutgesell et al., 2009). Although we have not directly measured fatty acid utilization here, it is probable that the up-regulation of liver TG-rich lipoproteins in blood are responsible for delivering lipids to the placenta and mammary gland. Given the increasing milk production during lactation, the total lipid transferred to the young via milk at peak lactation should be substantially greater than the amount of lipid transported during late pregnancy (Delongeas et al., 1997). However, this study did not observe any significant differences for apoB-100 levels between peak-lactation and late pregnancy, but apoB-100 levels quickly returned to non-reproductive levels following lactation.

High levels of circulating albumin during late pregnancy agree with prior work on rats at day 18 of gestation (Tam and Chan, 1977). However, a few studies focusing on humans display the opposite trend, with serum albumin dropping during pregnancy while other maternal serum proteins of hepatic origin increase (Maher et al., 1993; Murphy et al., 2002; Miida et al., 2008). The relative amount of NEFA transferred across the placenta varies by species (Jones, 1976; Leat and Harrison, 1980; Elphick and Hull, 1984). Rats have a hemochorial placenta, where the fetal epithelial and connective tissue and capillaries remain intact while bathed in maternal blood. Under these conditions, maternal NEFA transfer to the fetus may be higher in species with less invasive placentation (Koren and Shafrir, 1964).

During early pregnancy, maternal adipose accumulates, which is associated with hyperlipidemia and increased lipogenesis (Palacin et al., 1991; Herrera, 2002). During the last few days of gestation, these processes shift to reducing fat storage, enhancing lipolytic activity and accelerating breakdown of fat depots in adipose tissue and liver (Herrera et al., 1988). The rats in this study were sacrificed ~3 days before parturition. We cannot determine what percent of NEFA's were destined to adipose storage, the placenta, mammary gland, or cells that will oxidize the fatty acids to meet maternal energy demand. However, it is very likely that up-regulation of albumin represented an increase level of NEFA transport from adipose tissue to liver either to support the high energy demand of pregnancy in females or to be oxidized into ketone-bodies as an alternative fuel for the fetuses (Ghio et al., 2011). The reason for the drop in albumin after pregnancy is unclear, however, blood volume is reduced during lactation compared to pregnancy (Hytten, 1985; Wong et al., 2002). It is possible that a reduction in the time required for transport of NEFA resulted in less albumin is required. Moreover, albumin dropped back to control levels could also due to its multiple other physiological function other than NEFA transporter in blood (Rosenoer et al., 2014).

Intracellular lipid transport capacity in liver cells, as indicated by L-FABP_c_, was up-regulated during late pregnancy, and then returned to non-reproductive levels within a week of weaning. Previous work also indicates that L-FABP_c_ protein levels are high during pregnancy and lactation in the rat (Besnard et al., 1995). The pattern of L-FABP_c_mimics that of apoB-100. L-FABP_c_ is expected to reflect processes that will support lipid synthesis and modification by the liver. Thus, like apoB-100, we expect the up-regulation of L-FABP_c_ will transport lipids that will be stored by mothers or mobilized to the young. Prior studies have shown that homologs of L-FABP_c_ in other tissues, including heart (H-), intestinal (I-), and brain (B-) types of cytosolic fatty acid binding proteins, also to increase with circulating lipids and energy demand (Furuhashi and Hotamisligil, 2008; Zhang et al., 2015a,b), highlighting an important role for L-FABP_c_ in hepatic intracellular NEFA transport in helping reproductive females to meet the high energy demands of producing young.

Compared to non-reproductive females, both trans-membrane fatty acid transporters, FABPpm and FAT/CD36, were up-regulated in the liver of rats during late gestation and lactation. Few prior studies have focused on NEFA transport across the liver cell membrane during pregnancy (McManaman, 2014). Both transporters are expressed and up-regulated in the placenta in response to gestation (Dutta-Roy, 2000; Shennan and Peaker, 2000) and in the mammary gland in response to lactation (Bionaz and Loor, 2008). Interestingly Gutgesell et al. (2009) found the mRNA levels of FABPpm were reduced at day 15 of lactation relative to non-reproductive mice. It is possibly that FABPpm level were elevated at this time, the dropped in mRNA reflected the initiation of a down-regulation in fatty acid transport as pups begin consuming solid food and females near weaning. Additional data would be required to determine the relationship between mRNA and protein expression during the earliest stages of mammary regression.

By seven days after weaning, FABPpm, but not FAT/CD36, dropped to non-reproductive levels. Why FAT/CD36 would remain high is unclear. In addition to cooperating with FABPpm (Chabowski et al., 2007), FAT/CD36 appears to appears to play other important roles in cell membranes (Silverstein and Febbraio, 2009). FAT/CD36 appears also function as a scavenger receptor, which recognizes endogenously derived ligands, including apoptotic cells and oxidatively modified lipoproteins (Endemann et al., 1993; Ren et al., 1995). Consequently, elevated levels of FAT/CD36 could be responsible for apoptosis of liver cell and recognition of oxidation of lipoproteins during the post-lactation period. Moreover, recent data suggest that FAT/CD36 may also be regulated by thyroid hormone (Klieverik et al., 2009; Sinha et al., 2014), whereas post-lactation increase of thyroid hormone could also be responsible for FAT/CD36 levels remaining elevated (Hapon et al., 2003).

In this study, elevated FABPpm and FAT/CD36 was mainly due to uptake of NEFA into the liver. Increased levels of FABPpm and FAT/CD36 could be responsible for transporting NEFA into the liver from adipose tissue. Gutgesell et al. (2009) proposed a decrease of fatty acid uptake and β-oxidation by the liver is responsible for sparing fatty acids for milk production in the mammary gland. During lactation, fatty acid uptake, oxidation, and ketogenesis in liver declines (Whitelaw and Williamson, 1977; Gutgesell et al., 2009). While the drop in TG's and L-FABPc during lactation relative to pregnancy would likely with reduced fatty acid oxidation, the maintenance of FABPpm and FAT/DC36 suggest that fatty acid uptake by liver cells may not be reduced. Further research is needed to understand of how changes in liver function support reproductive demands.

## Conclusions

To our knowledge, this is the first study to simultaneously examine liver lipid uptake, transport and secretion during and immediately following reproduction. The lipid demands of gestation and lactation are supported at least in part by liver lipid secretion and transport capacities. Our data suggest that the secretion of lipids by the liver plays an important role during late gestation and lactation. Whereas, lipid uptake by the liver and within liver NEFA transport were up-regulated throughout reproduction. This elevation could serve as a mechanism of supporting the increase energy demand of female or supporting the high-energy demands of fetal development and milk production during reproduction.

## Author contributions

YZ, AK, and WH conceived the study and designed the experiments; YZ, CK, and HH collected the data; YZ analyzed the data; YZ, AK, and WH wrote the manuscript; YZ, AK, and WH interpreted data and revised the manuscript. All authors assume responsibility for the content of the paper.

### Conflict of interest statement

The authors declare that the research was conducted in the absence of any commercial or financial relationships that could be construed as a potential conflict of interest.
